# Design, Fabrication, and Optimization of a Printed Ag Nanoparticle-Based Flexible Capacitive Sensor for Automotive IVI Bezel Display Applications

**DOI:** 10.3390/s23094211

**Published:** 2023-04-23

**Authors:** Srinivasan Palanisamy, Muthuramalingam Thangaraj, Khaja Moiduddin, Hisham Alkhalefah, Panagiotis Karmiris-Obratański, Cheng Siong Chin

**Affiliations:** 1 Department of Mechatronics Engineering, Faculty of Engineering and Technology, SRM Institute of Science and Technology, Chennai 603203, India; 2 Department of Industrial Engineering, College of Engineering, King Saud University, P.O. Box 800, Riyadh 11421, Saudi Arabia; 3 Department of Manufacturing Systems, Faculty of Mechanical Engineering and Robotics, AGH University of Science and Technology, 30-059 Cracow, Poland; 4 Faculty of Science, Agriculture and Engineering, Newcastle University, Singapore 599493, Singapore

**Keywords:** Ag, bezel, optimization, printed sensors

## Abstract

Since printed capacitive sensors provide better sensing performance, they can be used in automotive bezel applications. It is necessary to fabricate such sensors and apply an optimization approach for choosing the optimal sensor pattern. In the present work, an effort was made to formulate interdigitated pattern-printed Silver (Ag) electrode flexible sensors and adopt the Taguchi Grey Relational (TGR)-based optimization approach to enhance the flexible sensor’s panel for enhanced automobile infotainment applications. The optimization technique was performed to derive better design considerations and analyze the influence of the sensor’s parameters on change in capacitance when touched and production cost. The fabricated flexible printed sensors can provide better sensing properties. A design pattern which integrates an overlap of 15 mm, an electrode line width of 0.8 mm, and an electrode gap 0.8 mm can produce a higher change in capacitance and achieve a lower weight. The overlap has a greater influence on sensor performance owing to its optimization of spatial interpolation.

## 1. Introduction

The integration of automotive electronics in vehicles plays a vital role on enhancing vehicle performance, safety, and ergonomics in order to meet customer expectations [1]. In cockpit electronics technology, considerable attention should be given to replacing conventional mechanical buttons and knobs into flexible touch panels to create a sense of futuristic technology and comfort for the passenger. Mechanical buttons may be constructed from a mix of plastic and silicon keypads, tactile switches, or rubber dome keypads. These are heavier, take up more space, noisier, more difficult to clean, and more vulnerable to damage from liquid and food spills, which may result in permanent damage. It is also difficult to incorporate such conventional technology into a futuristic cockpit with seamless and flawless profiles [2]. Human machine interface (HMI)-based light weight flexible electronics can reduce the weight of the infotainment bezel in automotive vehicles [3]. Nowadays, using flexible printed sensors (FPSs) is preferred among automotive fraternities owing to their the better production rates, flexibility, and ability to take on complex geometries [4]. Such flexible sensors can be easily manufactured using screen printing technology at a lower production cost and with good formability to make three-dimensional (3D) forms to achieve seamless integration within the cockpit [5]. For this reason, flexible printed sensors are mostly suited for use in in-mold electronics (IME)-based infotainment bezels [6,7]. In-vehicle infotainment system (IVI) bezels can be easily fabricated into 2D/3D forms through IME processes as per the ECE 21 automotive standard. These types of sensors can be classified into those using resistive and capacitive touch principles [8]. Since the resistive touch panel design does not support multi-touch gestures with null IME compatibility, it is suggested that capacitive-based flexible touch sensors be utilized for infotainment bezel applications. Table 1 explains the merits of capacitive sensors for use in automotive infotainment bezel applications [9,10,11,12]. Stretchable electronics, flexible batteries, and capacitive sensors, on the other hand, are relatively lesser industries with a high growth potential [13]. Expanding demand for energy-efficient, thin, and flexible consumer electronics, robust demand for next-generation flexible printed electronics, and the substantial cost reductions provided by printed electronics are driving market growth [14]. The preponderance of discrete electronic components are affixed on the printed conductive traces of a flexible substrate using conventional packaging techniques, such as surface mount technology [15].

**Figure 1:**
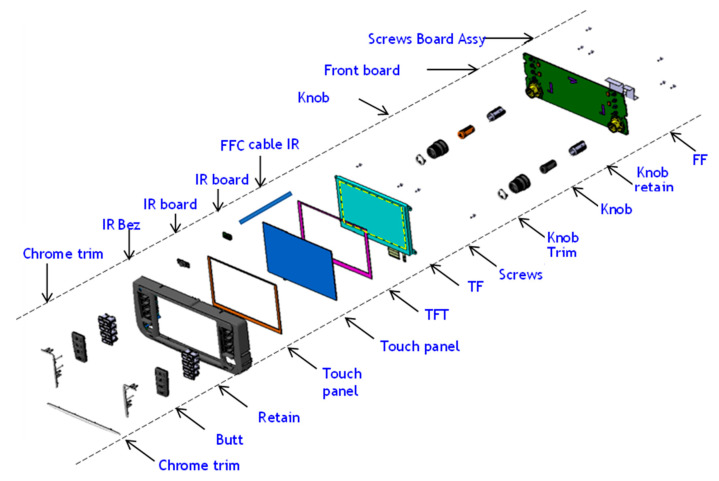
Typical infotainment exploded view with traditional technology.

**Figure 2:**
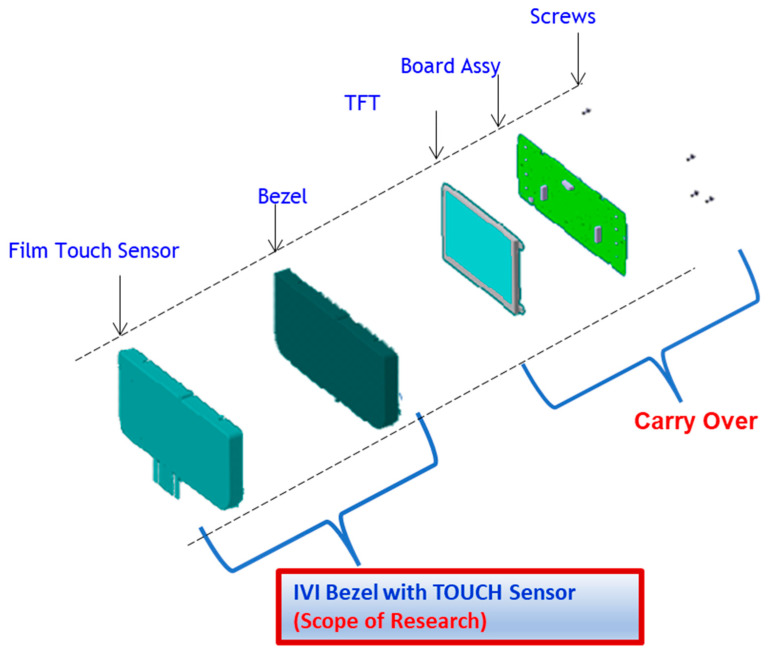
Proposed infotainment exploded view.

As illustrated in Figure 1, a traditional infotainment system consists of numerous technological components, including tactile switches, knobs, an encoder, and a display. Mechanical parts must be manufactured and maintained at a high level of quality across various locations around the world; manufacturing at a single location is not feasible due to the enormous complexity involved. In addition, logistics and the storage of parts are nightmares. The proposed infotainment system using FPSs, as depicted in Figure 2, is simple and has fewer parts, allowing for savings of space and weight, resulting in lower costs.

The capacitive-based touch screen sensors can be fabricated using insulated plastic sheets with conductive ink materials. The conductive ink pattern can be formed either with a continuous conductive line path pattern or a two isolated conductive electrodes-based interdigitated pattern [16,17]. Such flexible printed capacitive sensors are fabricated using two conductive ink pattern electrodes isolated by a dielectric medium. The nano-silver (Ag) conductive materials provide better sensitivity with a lower wear rate when compared to all other conductive materials for making FPSs in IVI bezel applications, owing to silver’s superior physical and electrical properties [18,19].

The flexible printed conductive layers are formed over a hard plastic layer to fabricate the final infotainment product. The spatial interpolation of the electrode structure in a capacitive touch panel has high significance when it comes to affecting the sensor’s performance, since the capacitance is directly proportional to the electrode overlap area [20]. The gap between electrode structures can also influence the sensitivity due its ability to change the capacitance. It is important to choose the optimal overlap area and distance between the electrodes to achieve better sensitivity and lower production costs [21].

Since the design and fabrication of flexible sensors involves more than one response, it is essential to implement multi-response optimization (MRO) to enhance such products [22]. It was found that Taguchi grey relational (TGR)-based MRO could give a better combination of optimal input factors with considerably better accuracy [23,24]. Flexible printed sensors (FPSs) are highly recommended by the automotive industry due to their higher production rate, flexibility, and capacity for complex geometries. Flexible sensors can be manufactured using screen printing with low production costs and the ability to form three-dimensional (3D)-shaped infotainment bezels for cockpit integration. Thus, printed flexible sensors are suitable for IME-based infotainment bezels.

In-vehicle infotainment system (IVI) bezels are manufactured in 2D and 3D using the IME process in accordance with the ECE 21 standard. Using screen printing technology, it is possible to rapidly produce flexible sensors for seamless cockpit integration with reduced production costs and excellent formability for developing three-dimensional (3D) objects. Consequently, flexible printed sensors are ideal for use in in-mold electronics-based infotainment periphery applications (IME). Conforming to the ECE 21 automotive standard, this allows for the in-vehicle infotainment system (IVI) bezel to be manufactured in 2D and 3D forms with relative simplicity using the IME method. The huge majority of Tier 1 suppliers have elected to transition the automotive industry to using multi-display modules which incorporate optical bonding, glass bending, and curved displays. Future user interfaces will incorporate curved glass displays. Three-dimensional surfaces offer infinite design possibilities.

From our detailed literature survey, we have inferred that only few research efforts have been dedicated to adopting optimization algorithms for designing and fabricating flexible printed capacitive sensors for the fabrication of IVI bezels. Only few research efforts have investigated the effects of the electrode parameters of sensor patterns on sensor performance. Hence, the present investigation has been undertaken. An effort was made to adopt TGR-based optimization algorithm to the fabrication of Ag-based FPSs and utilize this for IVI bezel modules in automobile industries.

## 2. Design of Silver Printed Flexible Capacitive Sensors

**Figure 3:**
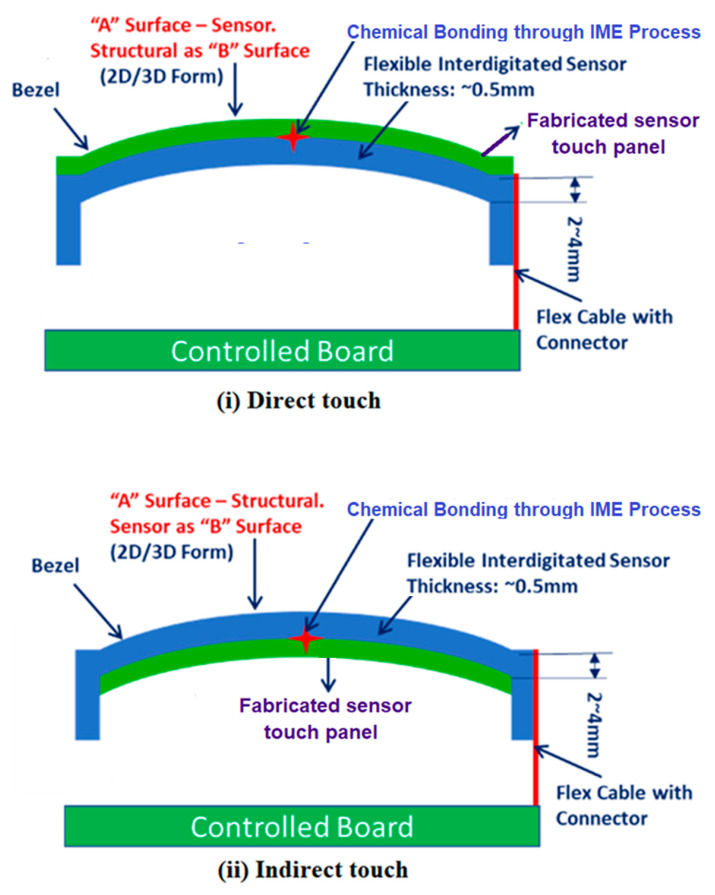
Schematic representation of infotainment bezel. (**i**) Conventional mechanical tactile button. (**ii**) Proposed flexible printed capacitive panel.

In this work, Ag nanoparticle (AgNP) conductive ink was used to make sensor electrodes (procured from Siltech Corporation INC, India) in FPS fabrication. These sensors were fabricated using the screen-printing process. Figure 3 shows a comparison of the schematic arrangement of a printed flexible electronic-assisted screen and conventional mechanical tactile buttons. The green curve in the figure indicates the fabricated sensor’s touch panel. In a direct touch fabrication, the touch panel is attached over the top surface. Hence, it will experience more tear and wear. However, in an indirect touch fabrication, the touch panel is attached below the transparent bezel. Hence, it will experience less tear and wear.

**Figure 4:**
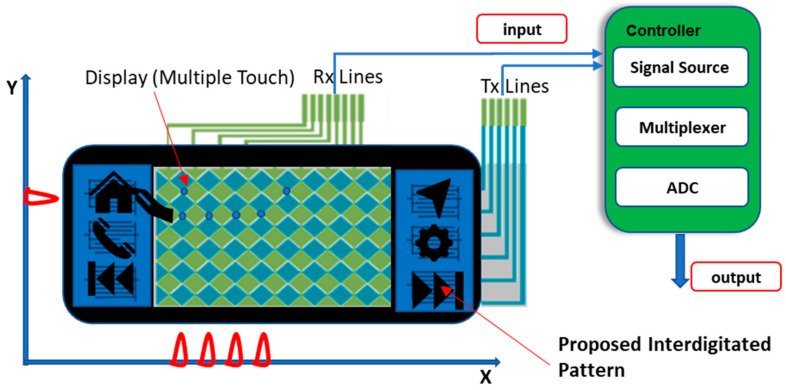
IME bezel with flexible capacitive sensor.

The weight of the infotainment system can be considerably reduced using the proposed printed sensor approach. The fabricated one-touch flexible printed capacitive sensors which use receiver (Rx) and transmitter (Tx) lines as keys can be framed over an IME bezel, as shown in Figure 4. Flexible printed capacitive touch sensors with Tx (Transmitter) and Rx (Receiver) electrodes have been developed using polycarbonate film. A capacitive sensing node can be considered to be a picture unit (pixel) at each intersection of the Tx and Rx lines, which is especially useful when viewed by a touch sensor to catch an image.

**Figure 5:**
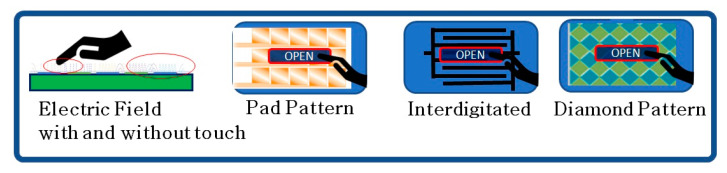
Types of flexible capacitive sensors.

Once the subsystem has sensed if a tactile incident in each node of the touch sensor has occurred, the touch sensor pattern in a multi-touch panel may be viewed as a touch image. The infotainment system can also include a host processor for receiving outputs from the panel processor and performing actions based on the outputs. The pattern can be pad, interdigitated, or diamond pattern in shape, as shown in Figure 5. In the present study, interdigitated pattern flexible printed capacitive sensors designed and were fabricated. When the passenger touches the capacitive key, the electric field is changed due to the spatial interpolation, as shown in Figure 3. The interdigitated pattern was selected, owing to its easy manufacturing process and considerable forming flexibility, based on advice from industrial experts [11].

### 2.1. Fabrication of Silver Printed Flexible Capacitive Sensor

**Figure 6:**
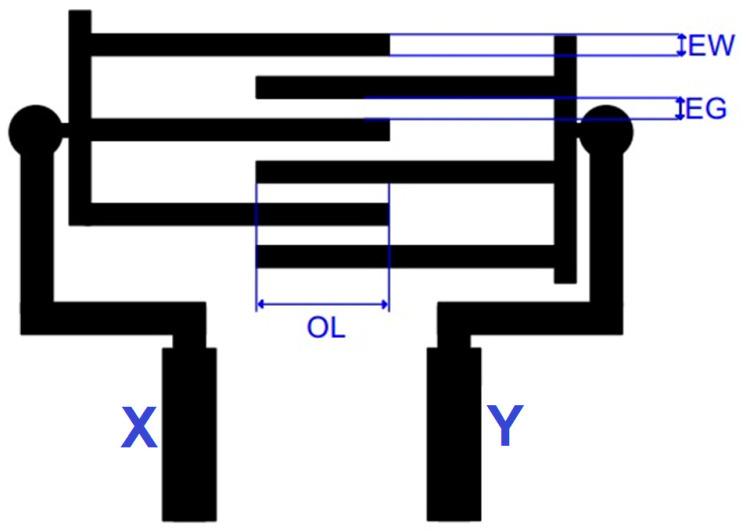
Design of flexible printed capacitive sensor.

An interdigitated comb pattern-based flexible capacitive touch panel with a size of 25 mm × 15 mm was designed and fabricated, as shown in Figure 6 [25]. Two isolated conductive electrodes (X and Y) were printed with a thickness of 20 µm over polycarbonate sheets with a thickness of 375 µm. An Ag ink particle was selected, owing to its electrical conductivity and opaque characteristics. A Lexan 8010 polycarbonate sheet (Sabic Innovative Plastics from the USA) was chosen as the plastic substrate, due to its better thermal resistance and processability, along with its higher transparency for use with IME.

**Figure 7:**
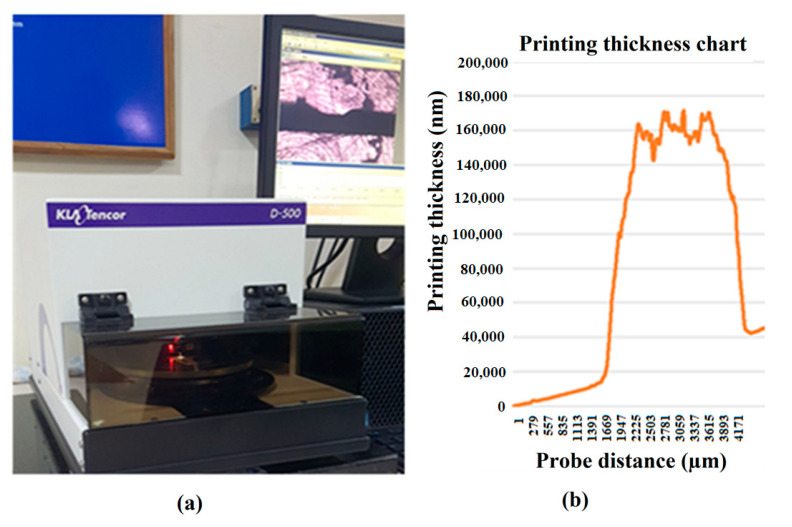
Fabrication process for flexible printed capacitance sensors. (**a**) Printing thickness verification using the KLA Tencor Alpha-Step D500 Stylus Profiler. (**b**) Printing thickness chart acquired using the stylus profiler.

Ag paste was applied above the photo-emulsion coating-based mesh screen frame (Saio Screen frame from India). The overlapping area of the electrode and line width of the electrodes are significant parameters for printed capacitive sensors, as per Equation (1).

(1) $$C=\frac{\varepsilon A}{D}\;,$$

where C is capacitance in farads, ε is the electrical permittivity in farads/meter, A is the capacitance area in m^2^, and D is the distance between electrodes in meters. The track thickness was measured at 20 µm using a Mextech CM-882. It was also verified using a KLA Tencor Alpha-Step D500 Stylus Profiler-based precision step height measurement, as shown in Figure 7. Then, a quality check was performed with a light inspection meter under 1000 lux.

### 2.2. TGR Optimization for Flexible Printed Sensors

The Taguchi methodology was used to design the experimental trials to be conducted for the experimental analysis in the present study. The steps in the TGR methodology are given below.

Step 1: Selection of orthogonal array (OA) [26].

OA ≥ D.E and(2)

(3) $$D.E=Q\left(P-1\right)+\;O{\left(P-1\right)}^{2\;}+1,$$

where D.E is the degree of freedom, Q is the number of independent variables, P is the number of their levels, and O is the number of interactions.

Step 2: Computation of the signal to noise (S/N) ratio and the normalized S/N (N S/N) ratio [16].

Step 3: Computation of the grey coefficient (GC)

(4) $${\mathrm{GC}}_{ni}=\frac{\left({\;\Psi }_{\min}+{\;\delta \Psi }_{\max}\;\right)}{\left({\;\Psi }_{ni}+{\;\delta \Psi }_{\max}\right)},$$

where Ψ*_ni_* is the grey coefficient for the *n*-th trial of the *i*-th dependent response and δ is the distinctive coefficient, which has a value between 0 to 1.

Step 4: Computation of the grey relation grade (G*_n_*).

(5) $$G_{n}=\frac{1}{M}\;\sum {\mathrm{GC}}_{ni\;,}$$

where *M* is the number of the performance measures.

Step 5: Confirmation experiment.

(6) $$G_{e}={\;G}_{f}-\sum \left(G_{h}-G_{f}\right),$$

where G*_e_* is the predicted GR grade, G*_f_* is the total average GR grade, and G*_h_* is the optimal average GR grade [27,28].

### 2.3. Measurement of Performance Measures

An attempt was made to find the optimal overlap distance (OL), electrode line width (EW), and electrode gap (EG) to achieve better printed sensing characteristics using TGR analysis. The Taguchi methodology was used to design the experimental trials to be conducted for the experimental analysis in the present study. The number of trial specimens to be used was decided to be nine, since the experimental was involved with two input factors, including OL (5 mm, 10 mm, and 15 mm), EW (0.5 mm, 0.8 mm, and 1.2 mm), and EG (0.5 mm, 0.8 mm, and 1.2 mm), along with three different levels [26]. The printing thickness was kept constant for all specimens.

## 3. Results and Discussion

### 3.1. Characterization of Fabricated Printed Sensors

**Figure 8:**
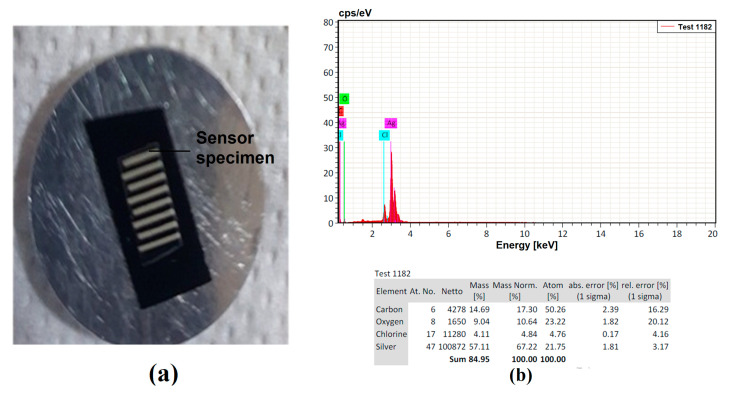
Printed sensor characterization using a scanning electron microscope. (**a**) Specimen in the chamber. (**b**) Elemental analysis using EDX.

**Figure 9:**
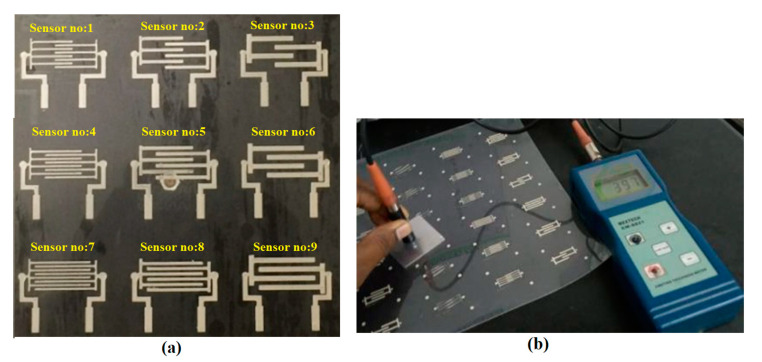
Fabrication process for the flexible printed capacitance sensor. (**a**) Fabricated sensor pattern. (**b**) Change in capacitance measurement while touching.

The flexible printed flexible capacitive sensors were fabricated using a screen printing process, and each sensor was cut using a shearing machine for further processing. A precision step height measurement utilizing a KLA D500 Stylus Profiler was used to confirm the results. The fabricated printed sensor tracks were characterized using a scanning electron microscope (SEM) and Olympus BX51 optical microscope (OPM). The elemental analysis was verified using SEM-assisted Bruker Energy Dispersive X-Ray Analysis (EDX), as shown in Figure 8. The major element, Ag, with other finer elements, was considerably observed with the sensor’s pattern to confirm the elements available in the sensor’s track. The remaining part indicated the binding agent components mixed with the Ag nanoparticles to form the ink for fabrication of the sensors. It was also observed that the electrode track was printed over the sheets with a uniform distribution. To avoid variations caused by different fabrication setups, the sensor design and the specimen were created in the same configuration. Application of the adhesive tape with uniform pressure applied with the fingers, followed by cautious removal of the tape with no jerking, was performed. A simple visual evaluation of the data may be sufficient to classify them according to the scale of the standard in question. The change in capacitance (ΔC) during touching (pF) and weight (mg) were considered as response parameters in the present study. The change in capacitance was measured using a Keysight U1733C—20,000 Counts Handheld LCR Meter with dual display, as shown in Figure 9. Table 2 shows the design of the experimental trials with the concerned performance measures in the present study. All trials were performed three times and the average of the values was considered to be the final value to improve the measurement accuracy, as shown in Table 2. The lower standard error shows the stability of the measured and computed values. The value of ΔC was considered to be the higher the better (HTB) and weight was considered to be the lower the better (LTB) in the characteristics trials to be conducted for the experimental analysis in the present study.

### 3.2. Computation of Optimal Process Factors

The signal-noise (S/N) ratios with normalized values (N S/N) were determined in Table 3 for the considered qualities of ΔC and cost. Since a higher change in capacitance can provide better sensitivity, it was considered to be the HTB. A lower weight can reduce production cost; hence, it was selected to be the LTB. Since the present study deals with both the HTB and LTB, the value of δ was selected to be 0.5 [27,28]. The grey relational coefficient for the individual response parameters and G*_n_* was computed and tabulated along with the rank, as shown in Table 4. It was inferred that specimen 7 can provide better performance measures.

The G*_n_* value and the rank are provided in Table 4. This rank indicates the optimal factor combinations. The overlap (15 mm), line width (0.8 mm), and electrode gap (0.5 mm) were established as optimum factors among the nine trials. Table 5 shows the average G*_n_* values for all the specimens fabricated. A larger value indicates an optimal factor. The optimal possible parameter combination was observed to be an overlap of 15 mm, line width of 0.8 mm, and an electrode gap of 0.8 mm, as shown in Table 6. A confirmation test was performed after the computation of the optimal factor combination optimization approach was completed to provide and enhance the performance of the FPS to inspect its confidence, as per Equation (6). The value of G*_n_* was increased by 1.9% from G*_e_* [26]. Hence, it was observed that the optimization approach could provide and enhance the performance of the FPS.

The metallization ratio (η) is a value which indicates the useful track utilization of the fabricated FPS. If the value is less than 0.5, it results in lower sensitivity and a higher weight. If the value is higher than 0.5, it results in higher sensitivity and a higher weight. Hence, 0.5 can be considered to be the optimal metallization ratio. The value of η can be computed using Equation (7):

(7) $$\eta =\frac{\mathrm{EW}+\mathrm{EG}}{\mathrm{EG}}.$$

**Figure 10:**
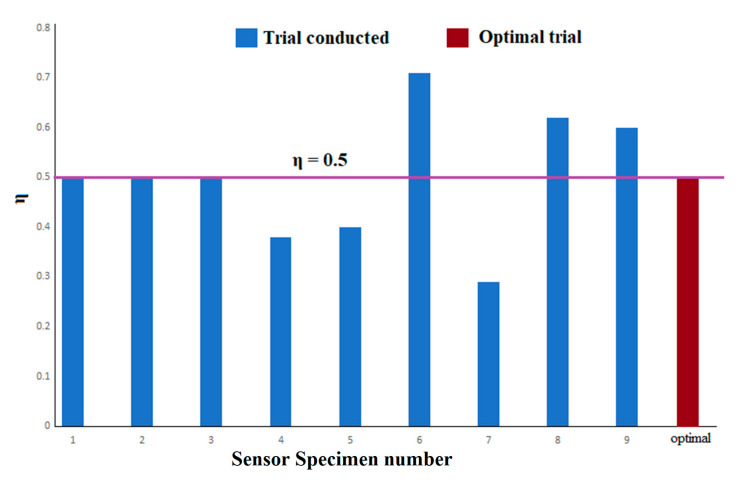
Effect of process parameters on metallization.

It was observed that trial 8 had an η of 0.62. The optimal combination derived from Table 7 had η as 0.5, as shown in Figure 10. Due to the effective utilization of metal tracks, this can provide better sensitivity with a lower weight of the fabricated printed flexible sensors.

**Figure 11:**
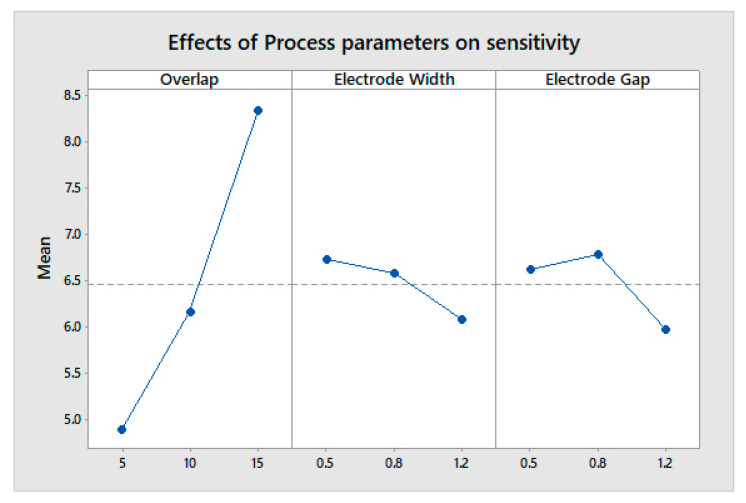
Effect of process parameters on sensitivity.

**Figure 12:**
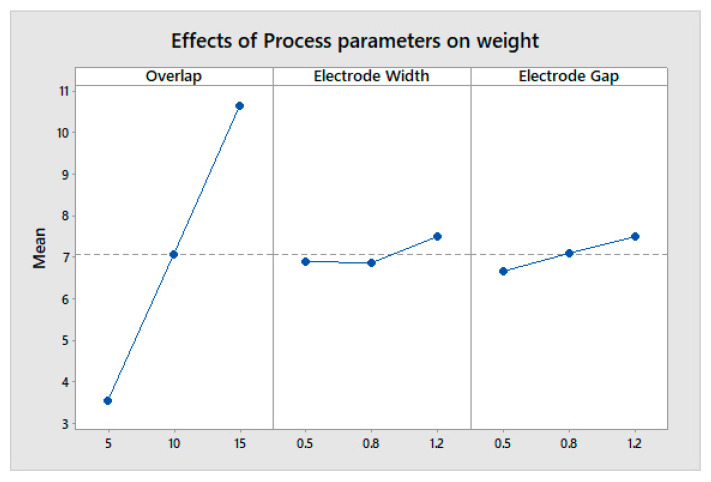
Effect of process parameters on weight.

**Figure 13:**
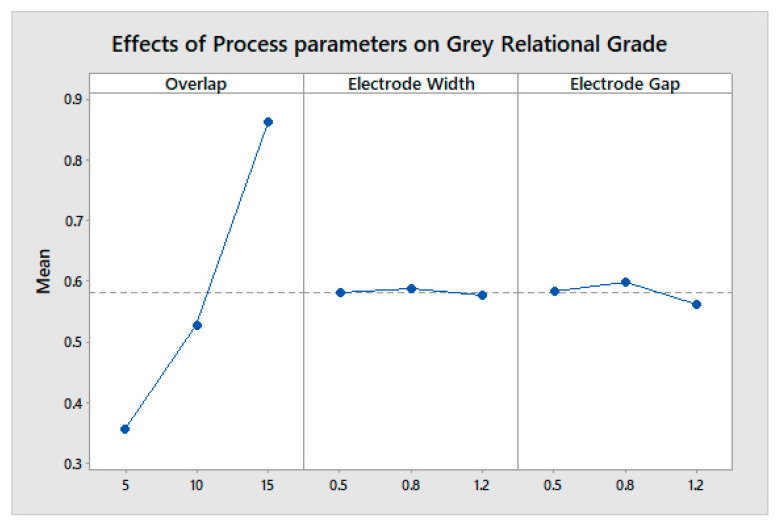
Effect of process parameters on the grey relational grade.

**Figure 14:**
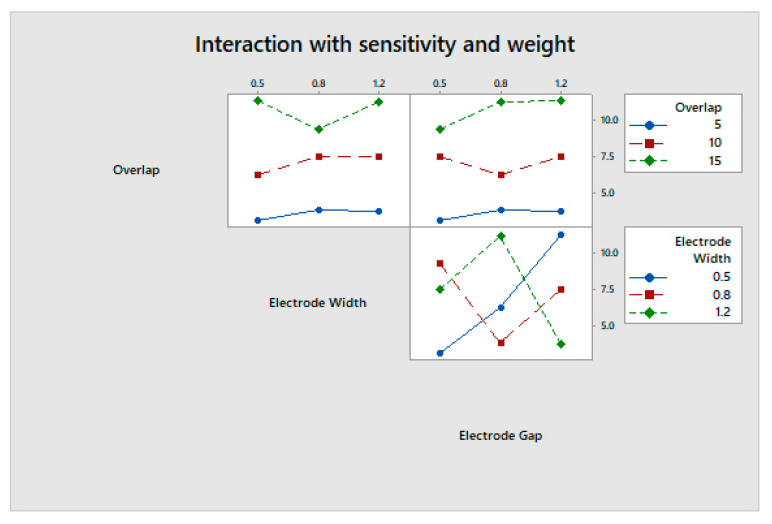
Interactions of process parameters on the grey relational grade.

The max-min value indicates the most influential factor in any system or process. The overlap area is the most influential in determining a higher change in capacitance and a lower weight, both individually and cumulatively, as shown in Figure 11, Figure 12 and Figure 13. The analysis was performed using a main effects plot. The deviation of the parameters from the horizontal mean line indicates a higher influence on output. In flexible printed capacitive sensors, the sensitivity is mainly determined by the capacitance developed in the touch panel. The capacitance is directly proportional to the overlap area and indirectly related to the square of the line gap, as per Equation (1). Since a higher overlap region creates a larger area of capacitance, the electric field intensity is considerably changed, owing to the spatial interpolation during touching. Hence, the overlap possesses a more influential nature when it comes to evaluating the performance measures in the design and fabrication process of flexible printer capacitive sensors. Figure 14 indicates the interactions between the process parameters for evaluating performance measures. The intersection of the line indicates the interaction between the process factors. It was observed that there were no interactions for the overlap area with the electrode line and the electrode gap. However, there were some interactions with the electrode line and electrode gap. This was also proved via the main effects plot.

### 3.3. Effect of Temperature on FPSs

**Figure 15:**
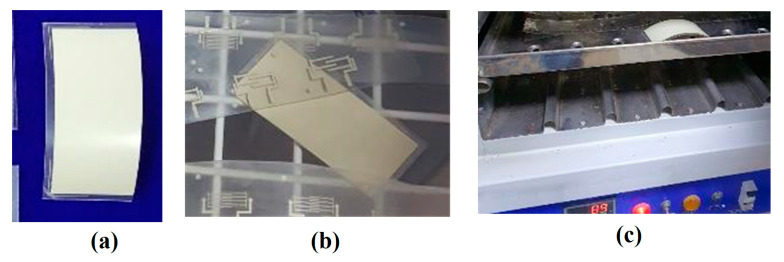
Thermal analysis of sensor specimens. (**a**) Fabricated specimen. (**b**) Specimen at −40 °C. (**c**) Specimen at 85 °C.

The worldwide automobile industry depends on research and development, design and analysis, material selection, and prototype testing. The vehicles must operate under different stresses, pressures, and climatic circumstances, such as temperature, rain, etc. The automotive industry must ensure that its products can withstand such rapid temperature changes without failure or material fatigue. This test was performed as per the IEC 60068 standard and OEM standards. The tests were conducted within a low temperature range of −40 °C for 42 h, then within a high temperature range of 85 °C for 48 h, as shown in Figure 15. A Hall effect measurement system (four-probe method) was utilized to compare the sheet resistance before and after the test. The FPSs exposure to heat lowered the sheet resistance, as shown in Table 8. This test ensured that the material can withstand temperature, humidity, and UV exposure to reduce breaking and consistency difficulties. Many OEM test specifications combine temperature and humidity. Thermal testing ranged from −40 °C to 90 °C, humidity ranges from 50% to 95% RH, and temperature shocks range from −40 °C to 90 °C within 10 s.

**Figure 16:**
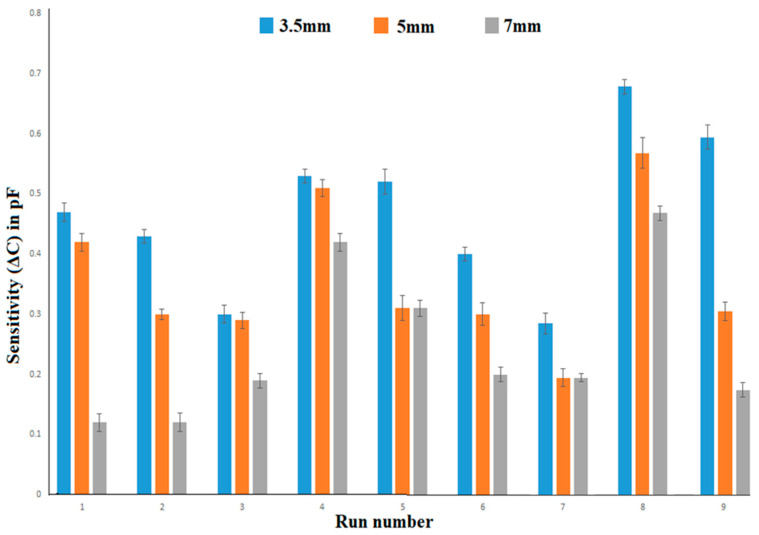
Comparison of sensitivity and forming depth.

The performance of the fabricated printed flexible capacitive sensor made using the screen printing approach was introduced to the forming process via vacuum technique. The change in sensitivity is illustrated in Figure 16 with respect to the forming depth of the sensor. All the trials were conducted three times and the average value was considered to be the final value in order to enhance the prediction accuracy. It was found that the error bar of the measurements could indicate the acceptable standard error. It was discovered that increasing the depth of the sensor could result in a decrease in ΔC, as shown in Figure 16. Since the electrode track could be stretched and elongated during the forming process, which could affect the interaction area between the electrodes and the gap between the electrodes, this could, in turn increase the resistance. Hence, a higher forming depth could result in a reduction of sensitivity in the FPS. It was found that the forming of the sensor pattern may affect the sensitivity. However, the optimal forming depth needs to analyzed for the effective design of the sensor pattern.

## 4. Conclusions

In this study, an endeavor was made to develop an interdigitated pattern printed Ag electrode flexible sensor and adopt the TGR-based optimization approach to enhance the flexible sensor’s panel for enhanced automobile infotainment applications. The optimization technique was performed to derive better design considerations and analyze the influence of the sensor’s parameters on changes in capacitance during touching and on production cost. The following conclusions were derived based on observation:

➢ The Ag nanoparticle conductive track-based interdigitated pattern with polycarbonate printed sensors offers considerable sensing ability.
➢ The interdigitated design with an overlap of 15 mm, electrode line width of 0.8 mm, and electrode gap of 0.8 mm produces a higher change in capacitance and a lower weight.
➢ The overlap is more influential to sensor performance, owing to its optimal spatial interpolation.
➢ Further research endeavors can be made which consider different production processes and optimization algorithms for FPSs.

## Figures and Tables

**Table 1:** Touch technology comparison.

Technology	Resistive	Projected Capacitive (PCap)	Surface Wave Acoustic (SAW)	Infrared
Method	Mechanical	Electrical	Acoustic	Optical
Principle	Mechanical pressure to activate	Electric field	Ultrasonic	Interruption
Light Transmittance	Max 70%	>88%	90–92%	92–99%
Resolution	Moderated	High	High	Low
Detection of Sensor	Volt	Capacitance	Delay in time response	Presence of light
Response Time	<10 ms	<15 ms	10 ms	<20 ms
Activation Force	10–100 g	Forceless	10–100 g	Forceless

**Table 2:** Computation of the sensor performance measures.

Printed Sensor Number	OL (mm)	EW (mm)	EG (mm)	ΔC (pF)	Weight (mg)
1	5	0.5	0.5	5.3	3.12
2	5	0.8	0.8	4.79	3.767
3	5	1.2	1.2	4.59	3.744
4	10	0.5	0.8	7.31	6.24
5	10	0.8	1.2	5.77	7.492
6	10	1.2	0.5	5.4	7.488
7	15	0.5	1.2	7.56	11.287
8	15	0.8	0.5	9.17	9.36
9	15	1.2	0.8	8.26	11.232

**Table 3:** Computation of the N S/N ratios.

Printed Sensor Number	S/N Ratio		N S/N Ratio	
	ΔC	Weight	ΔC	Weight
1	14.4855	−9.8831	0.2078	0.0000
2	13.6067	−11.5199	0.0616	0.1466
3	13.2363	−11.4667	0.0000	0.1418
4	17.2783	−15.9037	0.6724	0.5391
5	15.2235	−17.4920	0.3306	0.6813
6	14.6479	−17.4873	0.2348	0.6809
7	17.5704	−21.0516	0.7210	1.0000
8	19.2474	−19.4255	1.0000	0.8544
9	18.3396	−21.0091	0.8490	0.9962

**Table 4:** Computation of the grey relational grade.

Specimen Number	Grey Coefficient		G*_n_*	Rank
	ΔC	Weight		
1	0.3869	0.3333	0.3601	7
2	0.3476	0.3694	0.3585	8
3	0.3333	0.3681	0.3507	9
4	0.6042	0.5203	0.5623	4
5	0.4276	0.6107	0.5191	5
6	0.3952	0.6104	0.5028	6
7	0.6419	1.0000	0.8209	3
8	1.0000	0.7745	0.8872	1
9	0.7680	0.9925	0.8802	2

**Table 5:** Computation of the average grey relational grade.

Factor	Level 1	Level 2	Level 3	Max–Min
OL	0.3565	0.5281	0.8628	0.5063
EL	0.5811	0.5883	0.5779	0.0104
EG	0.5834	0.6003	0.4349	0.1654

Average G*_n_* = 0.5681.

**Table 6:** Optimal design parameters.

Factor	Level	Values
Overlap	3	15 mm
Line width	2	0.8 mm
Electrode Gap	2	0.8 mm

**Table 7:** Effects of process parameters on metallization.

Printed Sensor Number	Overlap (OL)	Electrode Width (EW)	Electrode Gap (EG)	η (Metallization)
1	5	0.5	0.5	0.50
2	5	0.8	0.8	0.50
3	5	1.2	1.2	0.50
4	10	0.5	0.8	0.38
5	10	0.8	1.2	0.40
6	10	1.2	0.5	0.71
7	15	0.5	1.2	0.29
8	15	0.8	0.5	0.62
9	15	1.2	0.8	0.60
Optimal	15	0.5	0.5	0.50

**Table 8:** Temperature analysis of the FPS for automotive standards.

Performance of Sensor@Temperature Test (−40 °C and 85 °C)				
Sl. No.	Influencing Parameters	Results Comparison		Interpretation
		Before Test	After Test	
1	Performance of Sensor in terms of Sheet Resistance in Ohms	0.14	0.09	Sheet resistance has improved in terms of improved touch sensitivity

## Data Availability

The data presented in this study are available in the article.
